# Empathic concern and personal distress depend on situational but not dispositional factors

**DOI:** 10.1371/journal.pone.0225102

**Published:** 2019-11-14

**Authors:** Sarah Fabi, Lydia Anna Weber, Hartmut Leuthold

**Affiliations:** 1 Department of Psychology, University of Tübingen, Tübingen, Germany; 2 Universitätsklinikum Tübingen, University of Tübingen, Tübingen, Germany; University of Exeter, UNITED KINGDOM

## Abstract

Empathic concern and personal distress are empathic responses that may result when observing someone in discomfort. Even though these empathic responses have received much attention in past research, it is still unclear which conditions contribute to their respective experience. Hence, the main goal of this study was to examine if dispositional empathic traits or rather situational variables are more likely to evoke empathic concern and personal distress and how the two empathic responses influence motor responses. We presented pictures of persons in psychological, physical, or no pain with matched descriptions of situations that promoted an other-focused state. Approach-avoidance movements were demanded by a subsequently presented tone. While psychological pain led to more empathic concern, physical pain led to higher ratings of personal distress. Linear mixed-effects modelling analysis further revealed that situational factors, such as the type of pain but also the affect experienced by the participants before the experiment predicted the two empathic responses, whereas dispositional empathic traits had no significant influence. In addition, the more intensely the empathic responses were experienced, the faster were movements initiated, presumably reflecting an effect of arousal. Overall, the present study advances our understanding of empathic responses to people in need and provides novel methodological tools to effectively manipulate and analyze empathic concern and personal distress in future research.

## Introduction

When observing another person in distress, as in an emergency situation, an empathic response might be triggered that ultimately motivates us to act in a certain way (e.g., rush to help). Generally, the notion of empathy refers to our understanding and responding to the affective state of another person [1]. Often it is defined as the similarity between the emotional states of the observer and a target person, with the observer knowing that the target is the source of her or his own feelings [2,3,4]. While there is no agreed-upon definition, empathy is thought of as a multi-dimensional concept that encompasses different empathic phenomena [5]. The distinction between affective and cognitive empathy has largely prevailed in empathy research [6]. On the one hand, affective empathy is thought to refer to the experience of an affective state congruent with those of the observed person, including phenomena such as emotional contagion and affective resonance [5,7]. On the other hand, cognitive empathy refers to putting oneself in the shoes of the other, which is thought to depend on the effortful cognitive process of perspective taking [5,8], which some authors however consider a precursor or precondition of empathy [9]. Moreover, it is further important to distinguish between trait and state empathy, the former relating to a person’s general ability to show empathy and the latter to the transient empathic responses triggered in a specific situation.

Of special importance for the present work are two affective empathy-related concepts that take into account both the role of the self-other distinction in empathy [2,3] and the motivational consequences of empathy. These concepts are empathic concern and personal distress ([3,9,10,11]; for a critical evaluation of these concepts in relation to empathy, see [12]). Empathic concern, often also called sympathy, is an other-focused emotional response of sorrow or concern that results from the comprehension of the target’s emotional state but differs from it [4,5,13]. Personal distress is a self-focused aversive response that results from the apprehension of another’s distress and is similar to the target’s state (e.g., [4,9]). Importantly, the observer’s experience of such a specific empathic response is assumed to have certain motivational consequences on behaviour. For instance, it has been proposed that empathic concern leads to an altruistic motivation and helping behaviour in order to reduce the other’s suffering, whereas personal distress leads to an egoistic motivation to reduce the own unpleasant feelings [14,15]. Crucially, there appears to be a link between empathy and approach- and avoidance related motivations, the latter being related to the triggering of specific actions [5,16]. Thus, situational emotional concern might trigger approach behaviour (e.g., care, helping), whereas personal distress might give rise to avoidance behaviour (e.g., withdrawal).

In contrast to these elaborated conceptual assumptions concerning the two empathic responses, their empirical investigation is scarce until today. Thus, it remains unclear when humans experience more empathic concern and when more personal distress. In this work, it is hypothesized that the two empathic responses are differentially influenced by dispositional empathy and situational factors such as type of the presented pain and the affect experienced by the observer when encountering a person in pain. Moreover, it is unclear whether the altruistic and egoistic motivations underlying empathic concern and personal distress, respectively, differentially impact on behaviour. Here, we assume that such a biasing influence of the two empathic responses on the motor system can be revealed in a movement compatibility task demanding approach-avoidance movements.

Which specific empathic emotion is experienced by an observer in a given situation (situational empathy) is not necessarily strongly related to his or her general tendency to empathise with others in a specific way (dispositional empathy) [9]. More recently, [17] proposed that individual factors in interaction with situational factors determine whether the observation of another in need leads to empathic concern or personal distress. Examples for individual factors are empathic traits, but also abilities of emotion regulation, and the emotional background of the observer (i.e. depressive mood). Situational factors might be the context, the emotion, and the level of arousal induced by the situation. In order to measure situational empathic responses, researchers have frequently used rating scales including various adjectives that are either typically associated with empathic concern (warm, tender, moved, compassionate, sympathetic, soft-hearted) or personal distress (alarmed, upset, worried, disturbed, distressed, troubled, perturbed, grieved) (e.g., [10,18]). In the following, we refer to these rating scales as Empathic Response Scale. The most commonly used instrument to measure individual differences in trait empathy, namely the general disposition to feel empathic concern or personal distress for persons in pain, is the Interpersonal Reactivity Index (IRI) [19,6].

The few behavioural studies that have investigated the relationship between dispositional and situational empathic responses indicated only modest correlations. Thus, [20] assessed dispositional empathic concern and situational empathic concern in response to the Katie Banks tape that told a story about a girl who had lost her parents and was responsible for her younger siblings [21]. The correlation between dispositional and situational measures was moderate (*r* = .28). [22] applied the same instruments and a single videotape about deprived children to induce empathy. Again, their results revealed correlations between dispositional empathic concern and situational empathic responses (empathic concern: *r* = .35, personal distress: *r* = .24). Finally, using seven sad excerpts of a TV show that participants rated with regard to their experience of empathic concern, [23] found a similar correlation between dispositional and situational empathic concern (*r* = .26). These results accord with those of a recent meta-analysis that included also numerous unpublished studies [24], reporting a mean effect size of *r* = 0.35 for the association between dispositional empathic concern and the feeling state of “being moved” that the authors equate with the concept of empathic concern.

**Fig 1:**
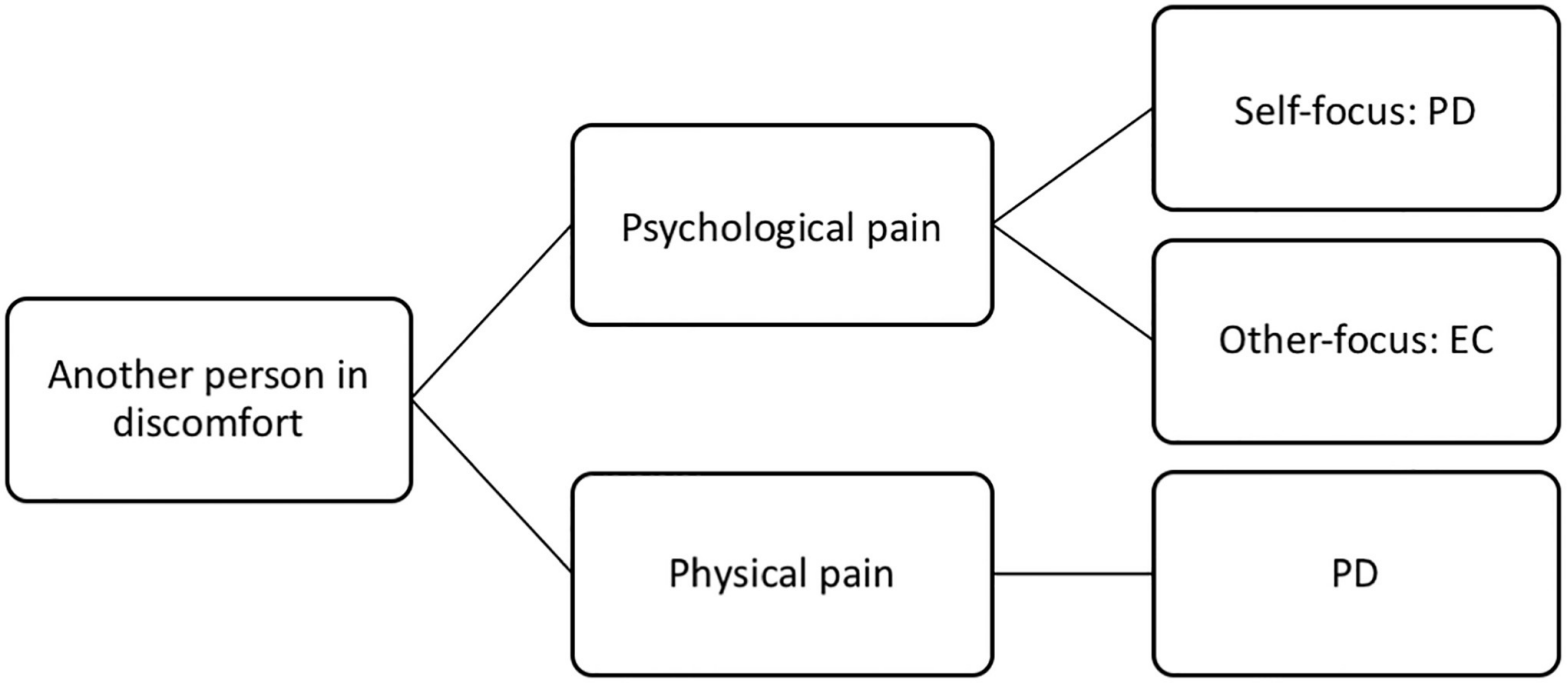
Empathic concern (EC) and personal distress (PD) as a result of physical and psychological pain combined with the focus of the observer.

The moderate correlations reported above suggest that other factors than just individual differences in dispositional empathy influence situational empathic responses. According to [10], the type of pain is crucial with regard to the specific empathic response elicited. That is, physical pain is assumed to evoke more personal distress in the observer, whereas psychological pain can evoke more personal distress or empathic concern, depending on the focus on the self or the other (see Fig 1) [17,25]. Specifically, a self-focused state, in which one projects oneself into the situation of the target, has been proposed to lead to higher personal distress, whereas an other-focused state, in which one focuses onto the target’s reaction, should lead to higher levels of empathic concern [1,10,26]. In support of this assumption, studies that induced psychological pain by presenting participants short stories such as that about Katie Banks found more personal distress under the instruction to maintain a self-focused state and increased empathic concern under the instruction to direct the focus onto the other [1,27]. A potential limitation of these studies is that they did not investigate the relation between dispositional and situational empathic responses.

The behavioural studies reporting moderate correlations between dispositional and situational empathic responses [20,22,23], did not explicitly control for the self- or other-focus participants adopted during the experiment. Consequently, it cannot be excluded that the focus adopted by participants varied, thereby influencing measures of situational empathic concern and personal distress. Moreover, to our knowledge, only [22] assessed the general tendency for positive and negative affect with the Positive and Negative Affective Schedule (PANAS) [28], even though other researchers [17] proposed that emotions and the arousal induced by a given situation play an important role in triggering empathic responses. For instance, [22] hypothesised that dispositional empathic concern is associated with experiencing both negative and positive dispositional affect because of persons’ general ability to accept their emotions instead of repressing them, whereas dispositional personal distress is associated with dispositional negative affect. However, they found that participants’ general tendency to experience negative affect correlated positively with their dispositional personal distress but not empathic concern. The tendency to experience positive affect, on the other hand, correlated negatively with dispositional personal distress but not with empathic concern. At least to our knowledge, the influence of situational positive and negative affect on the situational empathic responses has not yet been investigated.

Another issue concerns the fact that most studies relied on the spontaneous occurrence of situational empathic concern and personal distress in individuals following the presentation of only a single or very few tapes [20,22] or just a few video excerpts [23]. Given that situational empathy was assessed on so few occasions, it is also possible that the observed correlations are bound by potentially low reliability of the situational empathy score. Thus, it would be interesting to investigate whether the previously reported empathic effects replicate when participants are presented with other scenarios. Related to this point, so far research on empathic responses have not taken into account the variation of social stimuli due to sampling [29], leaving generalizations of findings across stimuli questionable. However, it is now possible to address this issue by conducting statistical analysis using linear mixed-effects (LME) modelling, in which both participants and stimuli are jointly included as random factors (cf. Method section). Together, it appears that the relationship between situational empathic responses and dispositional empathy is in need of further investigation, specifically by manipulating situational empathic responses more systematically and by using a large number of empathy-inducing stimuli. Thereby, more reliable situational empathy scores will be obtained and advanced statistical analysis becomes possible.

Also, as outlined earlier, it has not been investigated, at least to our knowledge, how the altruistic and egoistic motivations following from the two empathic responses translate into motor behaviour. Thus, previous studies analysing motor performance were mainly concerned with the general impact of empathy on information processing, reporting shorter response times (RTs) for empathy-inducing than control stimuli [30,31,32,33], but see [34]. Moreover, studies concerned with empathic concern and personal distress used too few trials to warrant an analysis of motor performance. Thus, by employing a larger number of empathy-evoking stimuli, it will become possible to investigate whether the two situational empathic responses differentially influence motor behaviour with regard to the following two aspects. First, according to [35], personal distress is accompanied by higher levels of physiological arousal than empathic concern. Since arousal is a variable known to enhance response speed [36], these two empathic responses might influence response time (RT) differentially. Second, empathic concern might lead to an altruistic motivation to reduce the target’s suffering, whereas personal distress might result in an egoistic motivation to reduce one’s own suffering by withdrawing from the situation [14,15]. We assume that these motivations manifest themselves in motor predispositions for approach or avoidance movements, respectively.

Whether such a biasing impact on the motor system is due to automatic affective versus controlled empathic processes is difficult to answer. However, whether the influence of empathic processes on the motor system is independent of the specific task goals can be examined. Thus, according to the feature-based definition of automaticity [37], an empathic influence on motor processing independent from the specific task goals could be conceptualised as “automatic” in this specific sense. In the movement compatibility task (see Method section for details) this can be accomplished if participants perform choice responses depending on another, empathy-unrelated, stimulus rather than on the empathic stimulus. To illustrate, [38] found faster withdrawal (key release) and slower approach movements (key press) to a visual go-/nogo-signal, when presented 500 ms but not when presented 100 ms after observing another person in physical pain, which presumably induced personal distress. On the one hand, this particular result demonstrates that empathic responses influence motor behaviour independent of task goals (automatically) in a movement compatibility task. On the other hand, it indicates that the time course of the underlying empathic or motivational process is presumably rather slow. Thus, it is conceivable that a cognitive rather than a fast automatic empathic process biases the motor system. Until now, at least to our knowledge, no study has investigated whether and how empathic concern and personal distress influence approach-avoidance motor behaviour in a goal-independent manner.

### Current study

In the present experiment, we were interested in the effects of different types of pain on empathic responses and the resulting motor behaviour while controlling for the potential impact of self/other focus. Since [10] claimed that psychological pain leads to empathic concern if the observer is taking an other-focused state, whereas physical pain should lead to personal distress independent of the focus, we presented pictures of persons apparently suffering from physical or psychological pain with a description of the situation (heart attack vs. death of the mother) promoting an other-focused state.

A primary aim of the study was to advance our understanding of the variables that influence the situational empathic responses, such as dispositional empathic traits or situational affect. More specifically, we tested the hypothesis that the general dispositional tendency to experience empathic concern and personal distress is positively associated with the actual experience of empathic concern and personal distress in a specific situation. Additionally, we hypothesised that empathic concern would be predicted by both high levels of positive and negative affect before the experiment, whereas personal distress would be predicted by high levels of negative affect only. This hypothesis is analogous to those of [22] for dispositional affect and dispositional tendencies in relation to the experience of empathic responses. Since the main focus of the present study was on investigating situational empathic responses and their triggers, we measured situational affect and not dispositional affect of the observer. Relatedly, several empathy researchers [5,39] have suggested that experiencing empathy is only possible in situations that one has already experienced oneself. Hence, we further tested the hypothesis that the experience of similar situations in the past leads to higher levels of empathic concern and personal distress.

As a second aim of the current study, we investigated empathy-related influences on motor performance. Assuming that situations in which a person experiences personal distress is more arousing than when experiencing empathic concern, we hypothesized that RT should be shorter for the former and to mainly decrease with increasing levels of individually experienced personal distress. Crucially, we assume that the altruistic approach motivation ascribed to empathic concern and the egoistic avoidance motivation related to personal distress [14,15] biases the motor system in a goal-independent manner. Accordingly, we hypothesized that approach movements should be faster in the psychological than the physical pain condition, whereas RTs for avoidance movements should be faster in the physical than the psychological pain condition.

## Method

In the following, we report how we determined our sample size, all data exclusions, all manipulations, and all measures in the study.

### Participants

We conducted a power analysis with the *R* package ‘pwr’ [40] to estimate the sample size required to detect a medium effect (f^2^ = 0.15) for psychological versus physical pain. To our knowledge, there are no comparable studies available on the basis of which effect sizes for the influence of type of pain on scores of empathic concern and personal distress could be estimated. Note that [1] found the manipulation of focus (self- vs. other) to produce rather large effects on these measures. Since it appears justified to assume that physical and psychological pain elicit differential effects on the respective measures, we opted for a medium effect size estimate. With the significance level set to α = .05, this analysis revealed a sample size of at least 54 participants to achieve a statistical power of (1-β) = .80, as recommended by [41]. 58 healthy adult students (44 females and 49 right-handed participants) from the University of Tübingen with mean age of 24.47 years (SD = 5.46, range = 18 to 47 years) voluntarily participated in the experiment for payment (8 Euros per hour) or course credits. They were informed in advance about the pseudonymous data recording and the anonymous long-term data storage, in accord with the General Data Protection Regulation (DGPR). The data of all 58 participants tested in this single experimental study were entered into statistical analysis; none of the data are published elsewhere.

### Apparatus

Stimulus presentation and response recording were controlled by a Mac Mini (Apple Inc.) running a MATLAB (The Math Works, Inc., Version R2015a) program using the Psychophysics Toolbox 3.0.12 [42,43] together with custom MATLAB routines. Participants sat in an electrically shielded, low-noise booth with ambient light at low level. A chin rest guaranteed a constant distance to the 1100 MB Samsung SyncMaster screen, on which materials were presented, with a resolution of 1280 × 960 pixels and a refresh rate of 60 Hz. Dimensions of the stimuli were 318 × 424 pixels at the beginning of each trial, but changed with movement. The response device consisted of a self-constructed metal box (“slider device”) measuring approximately 10 cm in height, 25 cm in width and 50 cm in length. The internals of the slider device consisted of rails along which a handle could be pushed/pulled (lengthwise with a total movement distance of 38 cm). A potentiometer was attached to the internal rails in such a way that the voltage output varied according to handle position. A software-based calibration routine converted the output voltage to cm and was calibrated such that 0 cm was the middle position, with values ranging from -18 to +18 cm. The internals of the device also contained an electromagnet that could be controlled online via software to prevent the handle from being moved. A force of ~ 150 cN was required to initiate the movement of the handle when the magnet was inactive.

### Materials

Auditory stimuli were sine waves of 400 and 800 Hz (60 dB) that were presented via headphones (Sennheiser, PX-100-II). Pictorial stimuli consisted of 117 pictures depicting persons in either emotionally neutral situations, and in situations in which they seemed to grieve or appeared to be haunted by strong pain in the chest (39 pictures for each type of pain: no pain, psychological pain, physical pain). Each picture depicted one person; both pain conditions showed the same number of racial ingroup (Caucasian) compared to outgroup targets (non-Caucasian). Pictures with physical pain and some of them with psychological pain were purchased from Fotolia (https://de.fotolia.com), a commercial picture platform. Further stimuli of sad persons were selected from the picture set used by [44]. The remaining pictures were selected from the International Affective Picture System [45]. The picture set will be provided by the authors upon request.

The different situations were picked because of their relatively high probability of experiencing them in daily-life and because pictures typically show stronger emotion-related effects than text stimuli [46]. Of course, it is conceivable that such pictures with a medium amount of pain evoke less intense empathic responses as compared to, for example, unusual pictures of emergency situations depicting mutilations. Additionally, written descriptions of the situation were provided (cf. S1 Table), which promoted an other-focus state followed by a statement that a person is sad because of the death of his or her mother or that the person experiences a strong pain in the chest (e.g., “Imagine yourself to be on a street facing a stranger who is obviously not feeling well. You ask him/her what has happened. He/she answers that he/she has just found out that his/her mother has died all of a sudden.”, “Imagine that you meet a new colleague at your office. All of a sudden, he/she complains about violent pain at the thoracic regions.”).

#### Pre-test

275 adult staff members and students of the University of Tübingen participated in a web-based pre-test of the stimuli. The picture items and their corresponding descriptions were presented together and were arranged in six stimulus lists. Each of the six 19–20 item lists consisted of three blocks with emotionally psychological, physical, or no pain pictures. Each list and hence each picture was rated by 45 to 46 participants on each of the seven dimensions (see Table 1).

**Table 1:** Dimensions of the picture rating.

Experienced empathic concern as the mean rating of six items (e.g., moved, sympathetic) of Batson et al. (1997)	1 (not at all) to 8 (very much)
Experienced personal distress as the mean rating of eight items (e.g., worried, upset) of Batson et al. (1997)	1 (not at all) to 8 (very much)
Arousal while watching the picture	1 (not at all) to 8 (very much)
State of the person depicted in the picture	1 (fine) to 8 (extremely bad)
Realism of the picture	1 (absolutely unrealistic) to 8 (absolutely realistic)
Fit between description of the situation and picture	1 (not at all) to 5 (very good)
Facilitation of imagining the situation by the description	1 (not at all) to 5 (very good)

In a pretest, pictures were rated on the seven dimensions presented on the left, with the response options at the right.

Mean rating scores are provided in Table 2. Analyses of variance (ANOVAs) with the between-subject factor type of pain (no pain, psychological pain, physical pain) and the random factor picture were performed with Bonferroni correction for multiple testing (alpha = 0.007). The results revealed significant main effects for all but the realism dimension (*F* (2, 114) = 4.15, *p* = .02), all *F*s (2, 114) > 15.36, all *p*s < .000001. To investigate the direction of the significant main effects, we performed post hoc Tukey tests that are reported in the next section.

**Table 2:** Results of the pre-test.

	Psychological pain	Physical pain	No pain	Psychological vs. physical	Physical vs. no pain	Psychological vs. no pain
**Observer’s state**						
**Empathic concern**	4.71 (0.64)	3.75 (0.45)	2.24 (0.65)	*p* < .001	*p* < .001	*p* < .001
**Personal distress**	3.02 (0.40)	4.01 (0.55)	1.63 (0.37)	*p* < .001	*p* < .001	*p* < .001
**Arousal**	4.75 (0.60)	5.16 (0.78)	2.81 (0.73)	*p* < .05	*p* < .05	*p* < .001
**Pictorial stimuli**						
**State of depicted person**	6.50 (0.77)	6.23 (0.78)	3.10 (0.87)	*p* = .37	*p* < .001	*p* < .001
**Realism**	6.12 (0.84)	5.72 (0.91)	5.58 (0.81)	--	--	--
**Description of situation**						
**Fit of description**	3.80 (0.56)	3.57 (0.66)	3.06 (0.49)	*p* = .19	*p* < .001	*p* < .001
**Facilitation of perspective taking by description**	3.89 (0.42)	3.62 (0.49)	3.35 (0.36)	*p* < .05	*p* < .05	*p* < .05

Mean rating scores on different dimensions for the three types of pain (with standard deviations in parenthesis) and *p*-values of Tukey tests.

Concerning the state of the observer, less empathic concern was provoked by the no-pain compared to the physical pain condition, *p* < .001, and by these two conditions compared to the psychological pain condition, *p*s < .001. Personal distress revealed lower values for the no-pain than the psychological pain condition, *p* < .001, and for these two conditions compared to the physical pain condition, *p*s < .001. Significantly lower arousal values were reported for the no-pain than the psychological pain condition, *p* < .001, which both differed from the physical pain condition, *p*s < .05, for which the arousal value was highest.

Regarding picture-related factors, the state of the depicted persons was rated as more positive in the no-pain than the psychological pain condition, *p* < .001, and the physical pain condition, *p* < .001, whereas scores in the psychological and the physical pain conditions did not differ, *p* = .37. Following Bonferroni correction, the ANOVA for realism of pictures did not reveal a significant condition effect, hence, no post-hoc tests were performed.

Concerning the description of the situation, the lowest rating for the match between description and situation was obtained in the no-pain condition, *p*s < .001, whereas pictures depicting persons suffering from psychological and physical pain did not differ in terms of fit of the description of the situation, *p* = .19. The facilitation of perspective taking with the aid of the description was larger in the psychological pain than the physical pain condition, *p* < .05, and both conditions showed larger values than the no-pain condition, *p*s < .05.

In conclusion, pictures were suited to provoke empathic concern and personal distress differentially. Physical pain pictures were given the highest arousal ratings in line with previous research [30]. Ratings of the state of the person displayed and the realism of the pictures were satisfactory, because they did not differ between the psychological and the physical pain condition but only with the no-pain condition. We therefore decided to use all picture stimuli, except for three pictures of each condition that were outliers on some dimensions and therefore selected to become filler items for the memory task. Descriptions of the situation did not differ in their fit to the pictures of the physical and the psychological pain condition.

#### Empathic Response Scale

The Empathic Response Scale [10] consists of six items per empathic response. Because of the frequent use of this scale in the present experiment, we shortened the scale to three items per empathic response based on a principal component analysis with Varimax rotation that was performed on the data of all six scale items included the pre-test. Two components were identified that explained more than 90% of the variance (49.9 and 40.8%, respectively). The selected items allowed to clearly differentiate between empathic concern and personal distress. The translated German version contained the following three adjectives for empathic concern: “mitfühlend” (compassionate), “bewegt” (moved), and “berührt” (tender) [component loadings: 0.956, 0.962, 0.969]. The three adjectives for personal distress were “beunruhigt” (worried), “alarmiert” (alarmed), and “ängstlich” (distressed) [component loadings: 0.960, 0.913, 0.925]. Mean empathic concern and personal distress scores were obtained by averaging the values of the ratings across the respective three adjectives.

#### Approach-avoidance task

The processing of the empathy-evoking stimuli was combined with a variant of the approach-avoidance task. This task has been shown to sensitively reveal the relationship between positively or negatively valenced items and specific approach vs. avoidance responses [47]. Using this task, previous studies have shown that emotional stimuli activate valence-dependent approach versus avoidance tendencies in a goal-independent manner [e.g., 48]. To assess such response tendencies, participants responded to an empathy-unrelated tone stimulus following 1000 ms after the presentation of the empathy-inducing picture. In the congruent conditions, meaning when the tone-instructed response matched the response that was activated by altruistic and egoistic motivations, participants were expected to respond faster. This procedure is similar to the one used by [38], in which participants responded to a visual stimulus following empathy-evoking pictures. They found a differential empathic influence on approach and avoidance movements for an ISI of 500 ms but not 100 ms, suggesting that empathic influences on the motor system need some time to develop. We therefore used a time interval of 1000 ms to assess whether slowly developing and longer-lasting empathic responses influence motor behaviour.

### Procedure

Before giving their informed consent, participants were informed that they would have to respond to a high- or low-pitched tone, while watching and memorizing pictures of persons in sad, neutral, or emergency situations. Furthermore, participants were informed that they could abort the experiment at any time without facing any negative consequences. Informed consent was obtained in a written manner and data was saved and analysed in a pseudonymous manner, with anonymous long-term data storage. Since there is no German provision that would require an ethics vote for behavioural investigations in healthy adults, we did not seek ethical approval for the present study.

At the start of the experiment, two practice blocks of six trials each were presented. Then, the experimental picture stimuli were presented in 18 blocks, each block consisting of a description of a situation and six pictures of the same category. As a result, each participant received 108 experimental trials, with each picture being presented once. In the memory task following each block, a total of nine old and nine new pictures (filler items) was presented.

In the current approach-avoidance task version, participants produced approach-avoidance movements to an affect-neutral auditory stimulus by pushing or pulling a lever depending on the tone’s pitch. A novel dynamic display arrangement was employed. Here, visual stimulus size was determined by the extent of the participant’s movement parameter. That is, when participants were to perform a pushing movement away from their body, the perceived depth position of the picture within a 3-D graphics scene moved away from the participant. Alternatively, when a pulling movement was required, the picture within the same scene appeared to move towards the participant. In contrast to static paradigms, this experimental setup allows one to disambiguate between the action and the outcome of this action. Specifically, within static paradigms, a movement away from one’s body can be internalized as either a push-away or a reach-to type of action.

**Fig 2:**
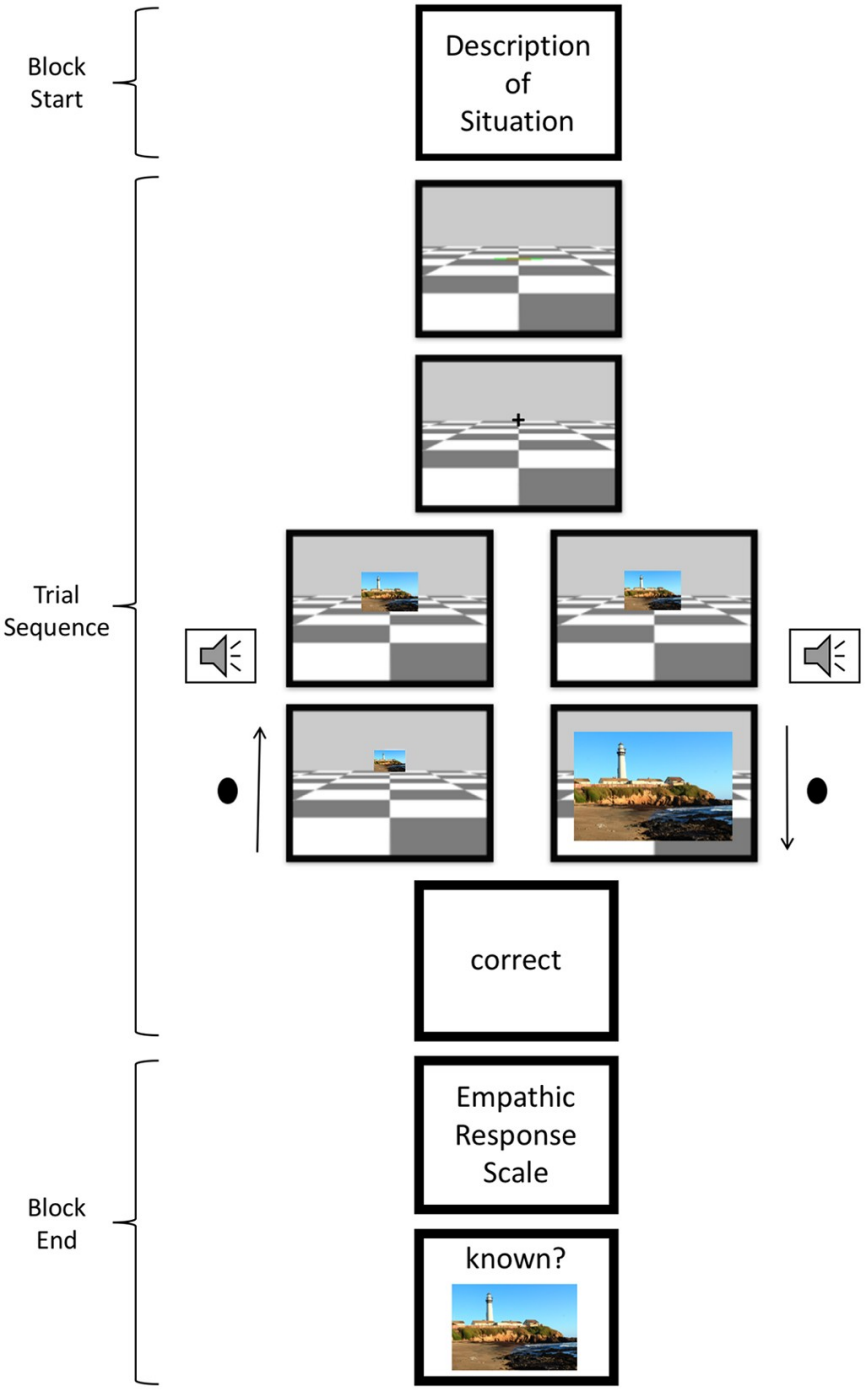
Schema of the procedure of one block: Presentation of the description of a situation, followed by six trials consisting of the start screen (1000 ms), fixation cross (1500 ms), presentation of picture, tone presentation 1000 ms after picture onset, approach vs. avoidance movement (up to 2000 ms) leading to an increase or decrease of the picture and 500 ms after movement offset, feedback presentation (1000 ms). At the end of the block, participants completed the Empathic Response Scale and the memory task. The picture was not part of the stimuli set but was selected for illustratory purposes.

The sequence of events within a single block is depicted in Fig 2. At the beginning of each block, participants were given a description of the situation, in which they were either meeting someone who was experiencing strong pain in the chest, who was grieving because of the death of the mother, or who was in an emotionally neutral situation. This was followed by the presentation of the six experimental picture trials. After each block, participants were asked to rate their empathic responses on the above introduced shortened version of the Empathic Response Scale [10]. Afterwards, they were shown a familiar or an unfamiliar picture and asked if it was part of the preceding block in order to direct their attention during the block onto the pictures.

Participants determined the start of each trial by bringing the slider into its start position. This position corresponded to +/- 1cm from the middle position as visually indicated on the stimulus display; once the slider was inside this region, the brake was applied. 1000 ms after bringing the slider to this position, a trial started with the presentation of a fixation cross for 1500 ms, followed by the display of the pictorial stimulus, thereby releasing the brake. After 1000 ms the tone was presented for 200 ms. Participants were to respond to the tone within 2000 ms following its onset. The picture disappeared 500 ms after movement offset and feedback was shown for correct, incorrect, too fast (movement onset < 200 ms), or too slow (movement onset > 2000 ms) responses at the centre of the screen for 1000 ms. A blank screen of 1000 ms followed. RT was defined as the time interval from picture onset to the time point when the slider reached a position 0.2 cm away from the actual starting point.

The order of physical pain, psychological pain, and no-pain conditions was randomised across participants with the constraint that approach and avoidance movements occurred equally often for each picture and that two consecutive blocks were always of the same condition. The mapping of tone pitch to movement direction (push/pull) was balanced across participants. In half of the blocks, the memory task consisted of a picture of the previous block, in the other half a new picture (filler item) was presented.

At the beginning and at the end of the experiment, we asked participants to complete the PANAS in order to assess situational positive and negative affect [28, 49]. At the end of the experiment, following the PANAS, participants were additionally asked if they had ever experienced a loss of a close relative or a heart attack of a close person. After answering the questions about their past experiences, they finally completed the German version of the IRI [19], the Saarbrücker Persönlichkeitsfragebogen (SPF) [50], as a measure of dispositional empathy.

### Data analysis

We analysed the influence of the different variables on empathic concern and personal distress scores using LME modelling [51] with the aid of R and the lme4 package [52]. In LME modelling, the sampling of participants and items (photographs in our case) can be taken into account by including participants and items as random effects in a single model [29,51]. Besides other benefits of the LME modelling approach, it allows generalization of results both across participants and items [29,51]. In the present study, as random effects we entered random intercepts for subjects and items and a by-subject random slope for type of pain. A further by-item random slope for type of pain was not included, because each photograph was presented only in one of the pain conditions, hence, being nested within type of pain. For empathic concern, we included in the full model as fixed effects dispositional empathic concern and dispositional personal distress, type of pain, positive, and negative affect before the experiment and excluded them in a stepwise manner. For personal distress, the full model included as fixed effects dispositional empathic concern, dispositional personal distress, type of pain, positive, and negative affect before the experiment, excluding them in a stepwise manner. After every step, models were compared in order to determine the best-fitting model.

In order to directly compare the influence of type of pain on the two empathic responses, a new dependent variable was calculated (difference score) and entered into a repeated-measures ANOVA including the factor type of pain with levels psychological and physical pain. More specifically, in a first step, the mean empathic concern and personal distress values of the neutral conditions were subtracted from those of the two other pain conditions for each participant to adjust for scale differences. In the second step, the resulting personal distress scores were subtracted from those for empathic concern. The resulting difference scores were entered as dependent variable into the repeated measure ANOVA.

For RT, we applied LME modelling. As random effects, we entered random intercepts for subjects and items and a by-subject random slope for type of pain. As fixed effects we included in the full model situational empathic concern and personal distress, as well as the interaction of Movement Direction (push vs. pull) × Type of Pain (psychological vs. physical).

The significance level was set to alpha = .05 and Bonferroni-corrected post-hoc tests were conducted.

## Results

### Descriptive results

#### Response accuracy

Mean response accuracy to the tone trials was high (98.12%; range = 84.25–100%). Participants also followed the instructions to process the pictures, as indicated by the high accuracy in the memory task (94.44%; range = 77.78–100%).

#### Positive and negative affect

As compared to the start of the experiment, values on the positive affect scale were lower after the experiment (27.74 vs. 25.18), *t*(57) = 4.27, *p* < .001, and higher on the negative affect scale (11.90 vs. 13.36), *t*(57) = 4.98, *p* < .001.

#### Dispositional empathy

Dispositional empathy, as measured by the SPF, ranged from 32 to 58 (*M* = 43.58, SD = 5.76), with a mean dispositional empathic concern value of 15.03 (SD = 2.57) and a personal distress score of 11.74 (SD = 3.31).

### Influences on situational empathic responses

Measurements of situational empathic responses via the shortened version of the Empathic Response Scale [10] showed excellent internal consistencies (empathic concern: *Cronbach’s α* = .98, personal distress: *Cronbach’s α* = .92). Values for both situational empathic concern (*M* = 3.28, SD = 1.30) and personal distress (*M* = 2.63, SD = 1.07) ranged between 1 and 8. The two empathic responses were highly correlated, *r* = .78, *p* < .001.

In the following, the results of the LME analysis are outlined. For empathic concern, the best-fitting model with type of pain, negative, and positive affect as fixed effects is presented in Table 3. Excluding dispositional personal distress from the full model did not deteriorate the model fit, *χ*^2^(1) = 0.37, *p* = .54. Model fit was also not deteriorated by further excluding dispositional empathic concern, *χ*^2^ (1) = 0.42, *p* = .52. In contrast, further exclusion of either type of pain, negative, or positive affect reduced the model fit significantly, *χ*^2^ (2) = 73.60, *p* < .001, *χ*^2^(1) = 3.94, *p* < .05, and *χ*^2^(1) = 4.02, *p* < .05, respectively.

**Table 3:** Best-fitting model for empathic concern with type of pain, negative, and positive affect.

Empathic Concern ~ Type of Pain + Negative Affect + Positive Affect + (1 + Type of Pain|Subject) + (1|Item)	
***Fixed effects***	**Estimate (Std. Error)**
Intercept	-0.67 (0.73)
Psychological pain	2.47 *** (0.21)
Physical pain	1.74 *** (0.17)
Negative affect	0.11 * (0.05)
Positive affect	0.04 * (0.02)
***Random effects***	**Explained variance (Std. Dev.)**
Items	0.02 (0.13)
Subjects	0.73 (0.85)
Psychological pain	2.40 (1.55)
Physical pain	1.61 (1.27)

Linear mixed-effects model with type of pain, positive, and negative affect before the experiment as fixed effects and random intercepts for subjects and items as well as by-subject random slope for type of pain;

* *p* < .05,

*** *p* < .001.

For personal distress, the best-fitting model with type of pain and negative affect as fixed effects is presented in Table 4. Excluding dispositional empathic concern did not deteriorate model fit, *χ*^2^(1) = 2.20, *p* = .14, as well as the further exclusion of dispositional personal distress, *χ*^2^(1) = 0.68, *p* = .41. The model fit was also not deteriorated by the further exclusion of positive affect, *χ*^2^(1) = 0.07, *p* = .80, whereas the exclusion of type of pain and negative affect deteriorated model fit significantly, *χ*^2^(2) = 61.30, *p* < .001 and *χ*^2^(1) = 9.27, *p* < .01, respectively.

**Table 4:** Best-fitting model for personal distress with type of pain and negative affect.

Personal Distress ~ Type of Pain + Negative Affect + (1 + Type of Pain|Subject) + (1|Item)	
***Fixed effects***	**Estimate (Std. Error)**
Intercept	0.07 (0.45)
Psychological pain	1.02 *** (0.14)
Physical pain	2.39 *** (0.23)
Negative affect	0.12 ** (0.04)
***Random effects***	**Explained variance (Std. Dev.)**
Items	0.01 (0.11)
Subjects	0.38 (0.61)
Psychological pain	1.03 (1.02)
Physical pain	3.10 (1.76)

Linear mixed-effects model with type of pain and negative affect before the experiment as fixed effects and random intercepts for subjects and items as well as by-subject random slope for type of pain;

** *p* < .01,

*** *p* < .001.

#### Type of pain

**Fig 3:**
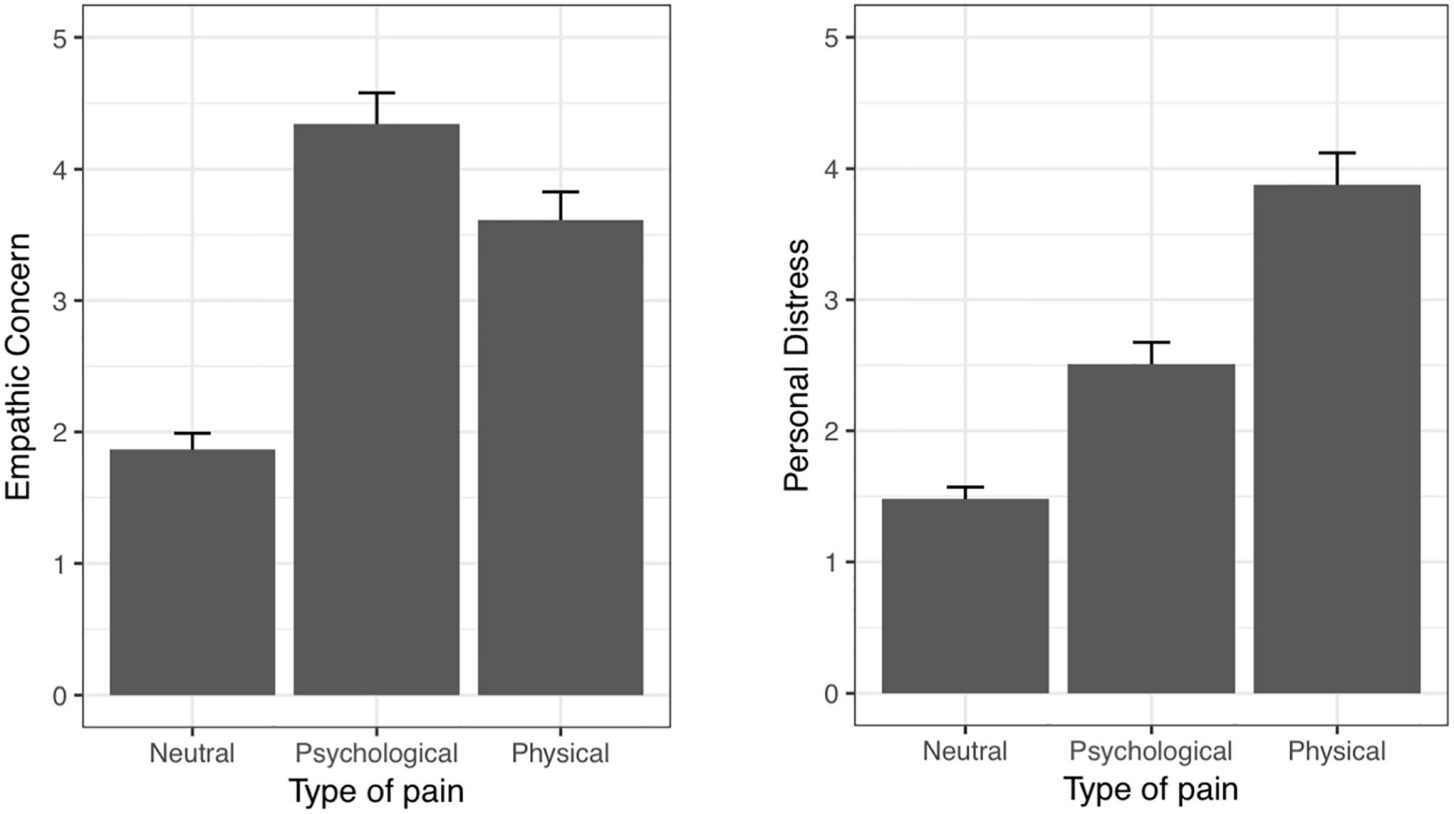
Situational empathic concern and personal distress scores as a function of type of pain.

The ANOVA of the mean difference scores of empathic concern minus personal distress corrected for scale differences (Fig 3) revealed a significant type of pain effect, *F*(1, 57) = 67.5, *p* < .001, *η*^2^ = .54. Post-hoc *t*-tests revealed that ratings of empathic concern were on average 1.44 (95% CIs [1.12, 1.77]) points higher than of personal distress in the psychological pain condition, *t*(57) = 8.92, *p* < .001. In the physical pain condition, the mean difference was negative (-0.65, 95% CIs [0.99, 0.31]) and as well significantly different from zero, speaking for lower empathic concern than personal distress ratings, *t*(57) = -3.82, *p* < .001.

#### Prior experience

45 of the participants had already experienced a loss of a close relative, whereas only 13 had experienced a heart attack of a close person. Those who had already experienced a loss reported less situational personal distress during the experiment than those who had not (2.49 vs. 3.08), *t*(25.91) = -2.09, *p* < .05, 95% CIs [2.16, 2.82], [2.59, 3.59]. However, whether or not participants experienced a loss of a close person did not differentially influence the amount of empathic concern (3.19 vs. 3.59), *t*(20.96) = -1.02, *p* = .32, 95% CIs [2.78, 3.58], [2.85, 4.32].

### Motor responses

**Fig 4:**
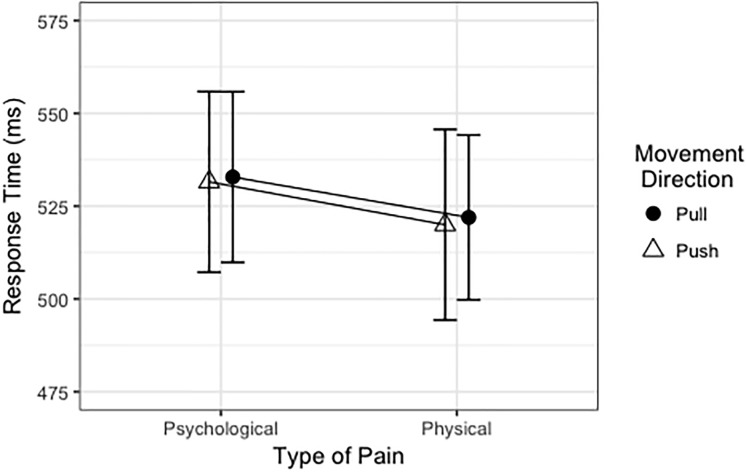
Response time in ms as a function of type of pain and movement direction.

For RT analyses, we excluded all incorrect response trials, trials with movement onset times shorter than 200 ms or movement offsets longer than 2000 ms (1.88%), as well as all partial error trials, in which slider movements started in the wrong direction but ended at the correct end point (3.77%). On average, participants responded 527 ms after tone presentation. The best-fitting model to explain RT with the fixed effects type of pain, situational empathic concern and personal distress is presented in Table 5. Excluding movement direction from the full model did not deteriorate the model fit, *χ*^2^(2) = 0.04, *p* = .98 (see Fig 4). Model fit was also not deteriorated by further excluding type of pain, *χ*^2^ (1) = 1.54, *p* = .21. In contrast, further exclusion of either situational empathic concern or situational personal distress reduced the model fit significantly, *χ*^2^ (1) = 11.15, *p* < .001 and *χ*^2^(1) = 14.50, *p* < .001, respectively.

**Table 5:** Best-fitting model for reaction time with situational empathic concern and personal distress.

Response Time ~ Situational Empathic Concern + Situational Personal Distress + (1 + Type of Pain|Subject) + (1|Item)	
***Fixed effects***	**Estimate (Std. Error)**
Intercept	590.21 (17.82)
Situational Empathic Concern	-9.48 ** (-3.36)
Situational Personal Distress	-8.29 ** (-3.89)
***Random effects***	**Explained variance (Std. Dev.)**
Items	176.60 (13.29)
Subjects	9428.30 (97.10)
Physical pain	529.00 (23.00)

Linear mixed-effects model with situational empathic concern and personal distress as fixed effects and random intercepts for subjects and items as well as by-subject random slope for type of pain;

** *p* < .01.

## Discussion

The aim of this study was to examine whether situational variables such as type of pain (psychological/physical) and affect influence empathic concern and personal distress, as well as how strongly these empathic responses are related to dispositional empathic traits. After assessing several variables such as affect, dispositional empathic traits, and situational empathic responses to pictures of persons in psychological, physical, or no pain, results showed the following pattern. Situational empathic concern and personal distress were influenced by situational variables but not by dispositional traits. Furthermore, the motivational consequences that are believed to follow from the two empathic responses have not been found to translate into approach and avoidance motor performance.

Before discussing the main findings concerning the occurrence of empathic concern and personal distress in detail, it is important to note the acceptable internal consistency for the shortened and translated version of the Empathic Response Scale [10], implying that this scale is a reliable instrument to measure situational empathic concern and personal distress. The present experiment differed from previous ones [20,22,23] with regard to the materials used to elicit empathic concern and personal distress. Crucially, as mentioned above, the individual values on both scales ranged between the lowest and highest possible value, implying that the current paradigm of presenting stimuli depicting quite probable daily-life situations is suited to provoke variable empathic responses across participants. Of course, mean empathic concern and personal distress scores did not reach extreme values but were in a similar intermediate range as in previous studies [14].

Most importantly, then, the present experiment succeeded in systematically manipulating the occurrence of the two situational empathic responses by varying the type of pain when participants held an other-focused state. As predicted, our results show that persons in psychological pain, because of the death of a close person, provoked significantly greater situational empathic concern than personal distress. On the other hand, persons with physical chest pain, as if during a heart attack provoked higher personal distress than empathic concern in the observer. At least to our knowledge, the current study is the first one investigating and supporting the assumption of [10] that physical pain evokes more personal distress in the observer, whereas psychological pain in combination with an other-focused state provokes more empathic concern.

It must be mentioned though that similar to previous research [14,22], we found the two responses to be highly correlated across participants. It therefore appears that people experience increasing levels of situational empathic concern with increasing levels of personal distress and vice versa. According to [14], there exist three reasons for this outcome: Empathic concern and personal distress are both elicited by situations with other persons in need. As a result, situational characteristics, such as pain intensity or the proximity to the person in need, influence both of them. Secondly, they are both affected by the general emotionality of the observer or even his or her willingness to report emotions. And finally, the meaning of the adjectives of the Empathic Response Scale are somewhat overlapping, thereby also contributing to the positive correlation.

Together, the present paradigm of presenting pictures depicting physical and psychological pain combined with a description of the situation inducing an other-focused state proofed promising in eliciting situational empathic concern and personal distress. Crucially, since participants performed choice responses to unrelated imperative tone stimuli, we consider it likely that picture content (type of pain), due to its task-irrelevant character, biased the motor system in a goal-independent manner. Moreover, the empathic responses elicited by the stimuli were reliably measured using a shortened Empathic Response Scale, thereby expanding the insights of previous studies that did not systematically manipulate the occurrence of empathic concern and personal distress [20,22,23]. In contrast to these studies, the present approach allowed to measure the spontaneous occurrence of situational empathic concern and personal distress to numerous stimuli while controlling for influences of the self- and other-focused state. As a result, it was also possible to treat both participants and stimuli as crossed random effects using a linear mixed-effect modelling analysis, thereby permitting generalizations across both participants and stimuli for the first time in field of empathy research. We propose that adopting the present methodological approach in future studies promises to reveal novel insights about situational empathic responses.

A second key finding concerns the association of situational affect and situational empathic responses. Here, the linear-mixed effect modelling approach extends previous work by incorporating various relevant variables while controlling for item-specific effects. These analyses showed that empathic concern is positively correlated with positive and negative affect, whereas personal distress is positively correlated with negative affect only. The missing link between positive affect and personal distress is in line with the suggestion that personal distress is experienced when observers are overwhelmed by negative emotions [53]. The current results are especially interesting since they appear to accord with the assumptions of [22] regarding the potential relationship between dispositional affect and dispositional empathic concern as well as personal distress, which so far has not been systematically investigated. Since Eisenberg and colleagues focused on the associations between dispositional variables and we on those between situational variables, future studies should measure both dispositional and situational affect and test which of these measures is associated with the occurrence of both dispositional and situational empathic concern and personal distress, while also taking into account the influence of affect.

A third major finding relates to the fact that dispositional empathic concern and personal distress did not reliably contribute to the explanation of the experience of situational empathic concern and personal distress, when dispositional (empathic traits) and situational variables (type of pain, negative, and positive affect) were included in the LME model. This outcome contrasts with those of moderate correlations between dispositional and situational empathy measures reported in previous studies [22,23]. These studies were not controlling for the influence of situational variables whereas the current study, at least to our knowledge, is the first one that jointly analyses state variables (affect, type of pain) and dispositional traits. Therefore, we assume that aspects of the situation are far more relevant to predict the occurrence of empathic concern and personal distress than individual differences in empathic traits. This particular insight has important implications regarding the examination of empathic responses more generally. For instance, recent studies concerned with the behavioural and neural correlates of empathic responses mainly focused on individual differences in dispositional empathic concern and personal distress [54,55,56]. However, they did not assess whether the empathy-evoking stimuli triggered individually different situational empathic responses as measured by the Empathic Response Scale, presumably because it is often implicitly assumed that dispositional empathy traits and situational empathic responses are strongly related to each other. At least in the light of the present data, this assumption is questionable and hence the above studies might provide a biased picture with regard to the neural correlates associated with empathic responses. Thus, future research should be concerned about this problem and additionally measure situational empathic responses when investigating neural and behavioural correlates of empathy.

A further relevant finding concerns the prior experience of participants. The general assumption is that empathy should be influenced by the observer’s experience [5,57,58]. Our results show that participants felt less situational personal distress during the experiment if they had already experienced the loss of a close relative than if they had not. This could be due to the knowledge that coping with difficult situations is possible.

Having established the relation between dispositional and situational variables, we further aimed at investigating whether the two emotions differentially influence motor processing. The results revealed that RT decreased with increasing intensity of both empathic concern and personal distress responses. This RT effect might be associated to the experience of higher arousal levels when facing a physical or psychological pain situation. However, contrary to our hypothesis, the physical pain condition did not result in shorter RTs than the psychological pain condition, although the evaluation of the arousal value of the pictures in our pre-test indicated a small but significant difference. However, since pictures in these two experimental conditions were generally rated as much more arousing than neutral pictures, their relatively strong arousal effect might have obscured the much smaller arousal effect triggered by the two empathic responses. Also, we did not find any differential influence of type of pain on RT for approach (pull) versus avoidance (push) movements. It must be noted that the tone was presented 1000 ms after the onset of the picture. Hence, it is possible that motor activations triggered by the empathy-evoking but response-irrelevant pictures might have already decayed at the time of tone onset. In this case, the present procedure would not have allowed to sensitively assess immediate automatic effects of the pictures on motor behaviour. On the other hand, previous results [38] suggest that it takes some time for the empathic influence on the motor system to develop. That is, we assume that introducing a delay between empathy-evoking picture and imperative tone signal allowed participants to thoroughly perceive the pictures and experience a potentially longer-lasting empathic response. Of course, similar to the study of [38], it would be informative to use a range of different short to long time intervals between picture and tone onset when determining the automatic motor consequences of the two empathic responses. Another potential limitation of the present study is that, even though the pre-test secured similarity on important dimensions, pictures have not been matched with respect to their luminance, the presented target’s sex, age, or attractiveness. Furthermore, future studies should have a look at the influence of demographic variables of the observer like age or sex by comparing equally distributed groups, since their influence is also not conclusively determined [59].

Moreover, while reducing external validity, future studies might also investigate whether the intensity of depicted situations differentially influence motor processing in terms of approach versus avoidance behaviour, for instance by presenting both high-intensity stimuli (e.g., pictures depicting mutilations) and the present more moderate everyday-life stimuli.

To conclude, this study investigated situational and dispositional correlates of empathic concern and personal distress jointly. This is especially interesting since in previous research, the situational approach [10,18] has been separated from the dispositional one [54,55,56]. Other studies investigated the relation between dispositional empathic traits and psychophysiological empathic responses such as brain responses or muscular activity, which cannot be equated with behavioural measures such as the Empathic Response Scale [60,61,62]. Our results, on the other hand, show that the novel multi-picture paradigm together with the shortened Empathic Response Scale are well-suited to elicit and to measure personal distress and empathic concern by manipulating in a task-irrelevant manner the type of pain presented. Crucially, at least under these conditions, personal distress and empathic concern are mainly driven by situational factors such as affect and type of pain rather than by dispositional empathic traits. This outcome has important implications for further research, suggesting that the research focus should be shifted from dispositional factors onto situational factors underlying empathic concern and personal distress. The multi-picture paradigm permitted the generalizability of findings across stimuli and participants using linear mixed effect modelling analysis. It also allowed the analysis of RTs, revealing faster responses for more arousing physical pain (personal distress) than psychological pain (empathic concern) items. Together, the present study advances our understanding of the commonalities and differences between these two empathic responses and also proposes novel methodological tools to effectively manipulate and analyze empathic concern and personal distress in future research.

## Supporting information

S1 Table Descriptions of the situations. Descriptions of the situations presented in German in order to induce an other-focus state; and their English translation.
(DOCX)

S1 File Data of the picture rating. (CSV)

S2 File R-script containing the data analyses. (R)

S3 File Data of the main experiment. (ZIP)
