# Supplementary material for: Empathic concern and personal distress depend on situational but not dispositional factors
Source: PLoS One. 2019 Nov 14;14(11):e0225102. doi: 10.1371/journal.pone.0225102 (PMC6855434; doi:10.1371/journal.pone.0225102)
Supplement: S1 Table — Descriptions of the situations presented in German in order to induce an other-focus state; and their English translation. (DOCX) [file pone.0225102.s001.docx]

**S1 Table. Descriptions of the Situations.**

|  | German | English |
| --- | --- | --- |
| No pain | Stellen Sie sich vor, Sie treffen am Arbeitsplatz auf einen neuen Kollegen bzw. eine neue Kollegin. Sie begrüßen sich gegenseitig. | Imagine yourself meeting a new colleague at the office. You say hello to each other. |
|  | Stellen Sie sich vor, Sie treffen Ihre neue Vermieterin bzw. Ihren neuen Vermieter. Sie begrüßen sich gegenseitig. | Imagine that you meet your new landlord. You say hello to each other. |
|  | Stellen Sie sich vor, Sie begegnen einem neuen Mitglied in Ihrem Verein. Sie begrüßen sich gegenseitig. | Imagine yourself facing a new member in your club. You say hello to each other. |
|  | Stellen Sie sich vor, Sie begegnen einem Fremden bzw. einer Fremden auf der Straße. Sie grüßen sich gegenseitig. | Imagine yourself facing a stranger on the street. You say hello to each other. |
|  | Stellen Sie sich vor, Sie begegnen einem Fremden bzw. einer Fremden in der Öffentlichkeit. Sie grüßen sich gegenseitig. | Imagine yourself facing a stranger in public. You say hello to each other. |
|  | Stellen Sie sich vor, Sie treffen Ihren neuen Nachbarn bzw. Ihre neue Nachbarin. Sie begrüßen sich gegenseitig. | Imagine yourself meeting your new neighbor. You say hello to each other. |
| Psychological pain | Stellen Sie sich vor, Sie haben zufällig ein Telefonat in der Öffentlichkeit mitgehört, in welchem der Person gerade mitgeteilt wurde, dass die Mutter überraschend gestorben sei. | Imagine that you have accidentally listened to a phone call in public in which the person was told that his/her mother has died all of a sudden. |
|  | Stellen Sie sich vor, Sie treffen ein neues Vereinsmitglied, dem es offensichtlich nicht gut geht. Sie fragen, was passiert ist und bekommen als Antwort, dass er bzw. sie gerade erfahren hat, dass seine bzw. ihre Mutter überraschend gestorben ist. | Imagine that you meet a new club member who obviously is not feeling well. You ask him/her what has happened and get as a response that his/her mother has died all of a sudden. |
|  | Stellen Sie sich vor, Sie haben einen Termin bei Ihrem neuen Vermieter bzw. Ihrer neuen Vermieterin. Sie bemerken, dass etwas nicht stimmt und fragen, was passiert ist. Er bzw. sie antwortet Ihnen, dass er bzw. sie gerade erfahren hat, dass seine bzw. ihre Mutter überraschend verstorben ist. | Imagine that you have an appointment with your new landlord. You notice that he/she is not feeling well and you ask what has happened. He/she responses that he/she just got to know that his/her mother has died all of a sudden. He/she responses that he/she just got the message that his/her mother has died all of a sudden. |
|  | Stellen Sie sich vor, Sie treffen Ihren Nachbarn bzw. Ihre Nachbarin und bemerken, dass er bzw. sie weint. Sie fragen, was passiert ist und bekommen als Antwort, dass er bzw. sie gerade erfahren hat, dass seine bzw. ihre Mutter überraschend gestorben ist. | Imagine yourself meeting your neighbor. You notice that he/she is crying. You ask him/her what has happened and he/she replies that his/her mother has died all of a sudden. |
|  | Stellen Sie sich vor, Sie treffen am Arbeitsplatz auf einen neuen Kollegen bzw. eine neue Kollegin, der bzw. dem es offensichtlich nicht gut geht. Sie fragen, was passiert ist und bekommen als Antwort, dass die Kollegin bzw. der Kollege gerade erfahren hat, dass seine bzw. ihre Mutter überraschend gestorben ist. | Imagine that you meet a new colleague at your office and notice that he/she is not feeling well. You ask him/her what has happened and he/she answers that your colleague has just got to know that his/her mother has died all of a sudden. |
|  | Stellen Sie sich vor, Sie begegnen einem Fremden bzw. einer Fremden auf der Straße. Dieser Person geht es offensichtlich nicht gut. Sie fragen, was passiert ist und bekommen als Antwort, dass er bzw. sie gerade erfahren hat, dass seine bzw. ihre Mutter überraschend gestorben ist. | Imagine yourself to be on a street facing a stranger who is obviously not feeling well. You ask him/her what has happened. He/she answers that he/she has just found out that his/her mother has died all of a sudden. |
| Physical pain | Stellen Sie sich vor, Sie begegnen jemandem auf der Straße, der plötzlich vor Ihnen zusammenbricht. | Imagine that you face a someone on the street who collapses all of a sudden in front of you. |
|  | Stellen Sie sich vor, Sie treffen am Arbeitsplatz auf einen neuen Kollegen bzw. eine neue Kollegin. Plötzlich klagt er bzw. sie über starke Schmerzen im Brustbereich. | Imagine that you meet a new colleague at your office. All of a sudden, he/she complains about violent pain at the thoracic regions. |
|  | Stellen Sie sich vor, Sie begegnen jemandem in der Öffentlichkeit, der plötzlich vor Ihnen zusammenbricht. | Imagine that you face someone in public who collapses all of a sudden in front of you. |
|  | Stellen Sie sich vor, Sie treffen Ihre neue Vermieterin bzw. Ihren neuen Vermieter. Plötzlich geht es ihm bzw. ihr immer schlechter. | Imagine that you meet your new landlord. All of a sudden he/she starts feeling worse and worse. |
|  | Stellen Sie sich vor, Sie haben einen Termin bei der Bank. Plötzlich klagt Ihr Berater bzw. Ihre Beraterin über starke Schmerzen im Brustbereich. | Imagine that you have an appointment at the bank. All of a sudden your counsellor complains about violent pain at the thoracic regions. |
|  | Stellen Sie sich vor, Sie sind bei Ihrem neuen Nachbarn bzw. Ihrer neuen Nachbarin zum Kaffee eingeladen. Plötzlich klagt der Mann bzw. die Frau über starke Schmerzen. | Imagine that your new neighbor invited you to coffee. All of a sudden, he/she complains about violent pain in the thoracic regions. |

Descriptions of the situations presented in German in order to induce an other-focus state; and their English translation.
